# No significant association between stable iodine intake and thyroid dysfunction in children after the Fukushima Nuclear Disaster: an observational study

**DOI:** 10.1007/s40618-020-01454-8

**Published:** 2020-11-18

**Authors:** Y. Nishikawa, C. Suzuki, Y. Takahashi, T. Sawano, H. Kinoshita, E. Clero, D. Laurier, G. Phan, T. Nakayama, M. Tsubokura

**Affiliations:** 1 Department of Internal Medicine, Hirata Central Hospital, 4, Shimizu-uchi, Kami-Yomogida, Hirata-mura, Ishikawa-gun, Fukushima 963-8202 Japan; 2 Department of Health Informatics, Kyoto University School of Public Health, Yoshida Konoe-cho, Sakyo-ku, Kyoto, 606-8501 Japan; 3 Department of Thyroid Surgery, Hirata Central Hospital, Shimizu-uchi, Kami-Yomogida, Hirata-mura, Ishikawa-gun, Fukushima 963-8202 Japan; 4 Department of Otolaryngology and Head and Neck Surgery, Graduate School of Medicine, Kyoto University, 54 Shogoin Kawahara-cho, Sakyo-ku, Kyoto, 606-8501 Japan; 5 Department of Radiation Health Management, Fukushima Medical University School of Medicine, 1, Hikariga-oka, Fukushima City, 960-1295 Japan; 6 The Institute for Humanistic Studies, Kamakura Women’s University, 6-1-3, Ofuna, Kamakura, 247-0056 Japan; 7 Health and Environment Division, Institute for Radiological Protection and Nuclear Safety, 92262 Fontenay-aux-Roses, France

**Keywords:** Iodine isotopes, Thyroid neoplasm, Radiation exposure, Fukushima nuclear accident, Disaster medicine

## Abstract

**Purpose:**

Stable iodine prophylaxis helps prevent childhood thyroid cancer in nuclear emergencies; however, there is limited information on its effect on thyroid function. This study aimed to examine thyroid function and autoimmunity among children and adolescents that took stable iodine after the Fukushima Nuclear Disaster.

**Methods:**

For this observational study, data were obtained from children and adolescents that underwent thyroid cancer screening at Hirata Central Hospital from April 2012 to March 2018. Participant characteristics, including possible hypothyroidism and hyperthyroidism, were compared between the prophylaxis and no-prophylaxis groups. Multivariable logistic regression models were used to assess for possible hypothyroidism, autoantibodies positive, and hyperthyroidism.

**Results:**

A total of 1,225 participants with stable iodine prophylaxis and 3,946 without prophylaxis were enrolled. Of those participants, blood samples were available for 144 and 1,201 participants in the prophylaxis and no-prophylaxis groups, respectively. There were 17 (11.8%) and 146 cases (12.2%) of possible hypothyroidism or autoantibodies positive cases in the prophylaxis and no-prophylaxis groups, respectively, and there were no cases and 3 cases (0.2%) of possible hyperthyroidism in those two groups, respectively. Multivariable analysis for possible hypothyroidism revealed no association between stable iodine intake and possible hypothyroidism or autoantibodies positive [odds ratio 0.716 (95% confidence interval 0.399–1.284)] (*p* = 0.262). We did not perform multivariable analysis for hyperthyroidism due to the limited number of cases.

**Conclusion:**

Significant adverse effects of stable iodine intake on thyroid function were not observed among children and adolescents 7 years after the Fukushima Nuclear Disaster.

**Electronic supplementary material:**

The online version of this article (10.1007/s40618-020-01454-8) contains supplementary material, which is available to authorized users.

## Introduction

Stable iodine prophylaxis is important for preventing thyroid cancer in nuclear emergencies along with other strategies such as evacuation, sheltering, and restricting the consumption of contaminated materials [1, 2]. Ingestion of stable iodine saturates the thyroid gland, thereby reducing the internal exposure of the thyroid by blocking the uptake of radioactive iodine and inhibiting iodide organification (Wolff-Chaikoff effect) [1]. Children and adolescents are at a higher risk of developing radiation-induced thyroid cancer compared to adults due to a range of physiological and behavioral factors [3]. Exhaustive distribution, timely and specific instructions for intake, and provision of pharmacological information should be addressed in order to adequately implement stable iodine intake for children and adolescents [4].

Even though it is a key strategy in cases of nuclear emergencies, reported adverse effects of stable iodine intake include allergies, skin rashes, swelling of salivary glands, and thyroid dysfunction among other effects [5–8]. Iodine-induced hyperthyroidism can be observed after treatment with iodine-containing drugs, such as anti-arrhythmic drugs or radiology iodinated contrast agents, and after iodine supplementation programs in regions of iodine deficiency when prescribed for a long period [9]. On the other hand, iodine-induced hypothyroidism may occur when iodine uptake does not escape the Wolff–Chaikoff effect, lasting around a few days in cases of acute iodine overload [1]. Hypothyroidism can also occur in fetuses after maternal iodine overload or in newborns during breastfeeding. These possible side effects during the neonatal period are concerning if not diagnosed and treated by hormonal therapy, as they may impair the long-term neurological and mental development of the child [9]. However, thyroid dysfunction can be confirmed through blood tests and has been well documented. Transient hypothyroidism could occur after the intake of stable iodine [7]. A Polish study after the Chernobyl accident revealed that there were transient functional changes (thyroid-stimulating hormone (TSH) increase) in 0.37% of neonates after iodine prophylaxis [8]. It was reported that serum T3 and T4 levels and antibodies in these children showed no significant differences between the prophylaxis and no-prophylaxis groups in Poland after the Chernobyl accident [8, 10]. Besides the Polish study, there is limited information on thyroid hormone levels amongst children and adolescents that took stable iodine in actual disaster cases.

On March 11, 2011, the Great East Japan Earthquake and tsunami occurred, followed by the Fukushima nuclear disaster. After radioactive substances, including radioactive iodine, were scattered, unintentional radiation exposure occurred among the residents [11]. The amount of radioactive iodine released after the Fukushima Nuclear disaster has been estimated to be about 10% of that after the Chernobyl accident (520 PBq vs. 5,300 PBq) [12]. As the estimated absorbed dose to the thyroid did not exceed 100 mGy in Japan [13], the implementation of stable iodine was not necessarily required [14]. However, seven local governments distributed stable iodine (potassium iodide tablets) to the residents, and four provided instructions on the intake of stable iodine [15]. Thus, stable iodine implementation occurred in specific local areas and not in the whole prefecture. Apart from the Fukushima Health Management Survey [16], Hirata Central hospital has been conducting voluntary thyroid screening since 2012 [17] in cooperation with municipalities inside and outside the Fukushima Prefecture. The screening program at Hirata Central hospital has been collecting data from blood and urine. Additionally, this screening program is the only setting that collects questionnaire-based information on whether stable iodine was taken after the disaster. Using the database of the Hirata Central hospital, the background and health status of the residents could be evaluated and classified based on the use of stable iodine prophylaxes.

The present study aimed to examine the thyroid hormone status and thyroid autoimmunity among children and adolescents who took stable iodine after the Fukushima Nuclear Disaster. This study provides important pharmacological information on implementing stable iodine prophylaxis among children and adolescents in cases of nuclear emergencies.

## Materials and methods

### Study design, setting, and participants

This observational study obtained its data from the thyroid screening program conducted at the Hirata Central hospital in the Fukushima Prefecture, Japan. Participants in this study were children and adolescents who underwent a thyroid screening at ≤ 18 years of age at the Hirata Central Hospital from April 2012 to March 2018 (the Japanese fiscal years 2012–2017). Participants who did not respond to the questionnaire regarding their intake of stable iodine or were taking medication for thyroid diseases at the time of screening were excluded from the study.

### Stable iodine distribution

The distributed doses of potassium iodide tablets were as follows: ~ 1 month old, 17 mg; 1 month–2 years old, 25 mg; 3–12 years old, 50 mg; and 13–39 years old, 100 mg.

### Thyroid screening at the Hirata Central Hospital

Hirata Central Hospital has been conducting voluntary thyroid screening since 2012. This screening program has been performed separately from the Fukushima Health Management Survey that followed the general population in Japan. Differences between the Fukushima Health Management Survey (FHMS) and Hirata Central Hospital (HCH) were observed with respect to participants who were residents of the Fukushima Prefecture (FHMS) or residents inside and outside of the Fukushima Prefecture (including the nearby Ibaraki Prefecture, etc.) (HCH) and *primary screening*, which included only ultrasound (FHMS) or ultrasound, urinary iodine, and blood sampling (recruitment of blood sampling is described below) (HCH). While ultrasound screening was targeted in all the examinees, blood samples were obtained only if the participants visited the hospital with their guardian.

### Independent application and school screening

There were two application methods: independent application and school screening. In cases of independent application, the residents directly applied for the screening at the Hirata Central hospital through the website or local community magazines. Participants from more than 50 municipalities applied through this method. In this setting, children and adolescents voluntarily visited the hospital, mostly with their guardians. Blood samples were collected for all subjects aged six years or older with written consent.

In the school screening, two municipalities were included. Participants were recruited from elementary (6–12 years old) or middle school (12–15 years old) (both included in Japan’s compulsory education system) on a voluntary basis after the Fukushima nuclear disaster. In one of the towns, it was observed that, in addition to the Fukushima Health Management Survey, thyroid cancer screening continued for primary and secondary school students in 2013, 2014, and every other year since 2015, as well as for people who voluntarily underwent thyroid screening at the hospital. Another town also provided thyroid cancer screening for school students but only during the fiscal years of 2014 and 2015. In the school screenings, only cases requiring further investigation underwent blood tests (findings such as nodules, large cysts, heterogeneity, etc.). During the first school screening, the students visited the hospital with the school teachers but without their guardians; only when the students required blood tests, they were they required to revisit the hospital with their guardians. Thus, there were differences in the number of blood tests between those who enrolled through independent application and school screening.

### Self-administered questionnaire

Before thyroid cancer screening, all guardians of the participating children or adolescents were asked to complete a self-administered questionnaire, which included information on whether the participants took stable iodine orally after the disaster. In the case of school screening, the questionnaire was distributed in the school. The guardians completed the questionnaires at home and submitted them at the time of the school screening. All questionnaires were collected at the hospital.

### Ultrasound screening

In the ultrasound screening, the volume of the thyroid and the thyroid screening categories were recorded. Volume for each of the side lobes was calculated using the following formula: Volume (mL) = Thickness (cm) × Width (cm) × Length (cm) × π/6. The total thyroid volume was obtained by summing up the volumes of both the lobes. The isthmus was not considered in the volume calculation [18].

### Data and variables

The data extracted from screening records included the ages of participants at the time of the examination, and at the time of the disaster, the sex, whether the participant was evacuated, and whether the participant took stable iodine orally after the disaster (as self-reported in the questionnaire). Areas were categorized into two based on whether or not the stable iodine prophylaxis was officially recommended by the local government [15]. Application methods included two categories (independent application or school screening). School screening targeting students aged 6–15 was performed in two municipalities (one town with data from 2013 and another town with data from 2013 and 2014). As for laboratory data, blood parameters [thyroid-stimulating hormone (TSH) (μIU/mL), free thyroxine (FT4) (ng/dL), free triiodothyronine (FT3) (pg/mL), thyroglobulin (Tg) (ng/mL), anti-thyroglobulin antibody (TgAb), anti-thyroid peroxidase antibody (TPOAb)], and urinary iodine (μg/L) were included. Through ultrasound screening, data on the volume of the thyroid were obtained.

### Criteria for thyroid dysfunction

Thyroid disorders were diagnosed based on the results of the blood tests and the guidelines for the diagnosis of thyroid disease by the Japan Thyroid Association [19]. In this guideline, the lower limit of TSH for diagnosing hyperthyroidism was documented as 0.1 ng/dL. For other parameters, higher and lower limits for thyroid hormone and thresholds for autoantibodies positive were based on the facility’s standard values (supplemental table). All the following examinations were performed by BML, Inc (Tokyo, Japan). Classifications were as follows: autoantibodies positive (TPOAb high or TgAb high); possible hypothyroidism (defined as the presence of TSH high or FT4 low); and possible hyperthyroidism (TSH $\le$ 0.1 μIU/mL and FT3 high and/or FT4 high).

### Statistical analyses

To assess whether thyroid dysfunction was associated with stable iodine prophylaxis, the following comparisons were made. All continuous variables except TSH, FT4, FT3, TPOAb, and TgAb were normally distributed. Normally distributed variables are presented as means and standard deviations, and non-normally distributed variables are presented as medians and interquartile ranges. Participants’ characteristics were compared between two groups: the prophylaxis group (residents who took stable iodine) vs. the no-prophylaxis group (residents who did not take stable iodine). Among participants who underwent blood tests, the values of test results, and the frequencies of subclinical hypothyroidism, antibodies, and hyperthyroidism were compared between the two groups (prophylaxis and no-prophylaxis). The significance of the difference between the two groups was assessed using an unpaired Student’s *t*-test for continuous variables and a chi-square test for categorical variables. Regression analysis was performed to further examine what factors observed after the disaster were associated with possible hypothyroidism or autoantibodies positive. The multivariable logistic regression model was used to identify factors associated with possible hypothyroidism or positive for autoantibodies. The following explanatory variables were included in the model: sex, age at the time of the disaster, years since the disaster, family history of thyroid diseases, school screening (or not), and iodine prophylaxis. Information on missing data is shown in each table. All cases with complete data were used in the model. The feasibility of a multivariable logistic regression model for possible hyperthyroidism was also considered. Statistical analyses were performed using R version 3.5.0 (https://www.r-project.org). All p-values presented are two-sided, and a *p* value of < 0.05 was considered statistically significant.

### Ethical considerations

This study was approved by the ethics board of the Hirata Central Hospital (2017-0321-2), the Ethics Committee of the Kyoto University Graduate School of Medicine (R1459), and the Fukushima Medical University Certified Review Board (30180). Formal informed consent was obtained from all participants (or their guardians in the case of participants aged < 20 at the time of the screening) included in this study.

## Results

**Fig. 1:**
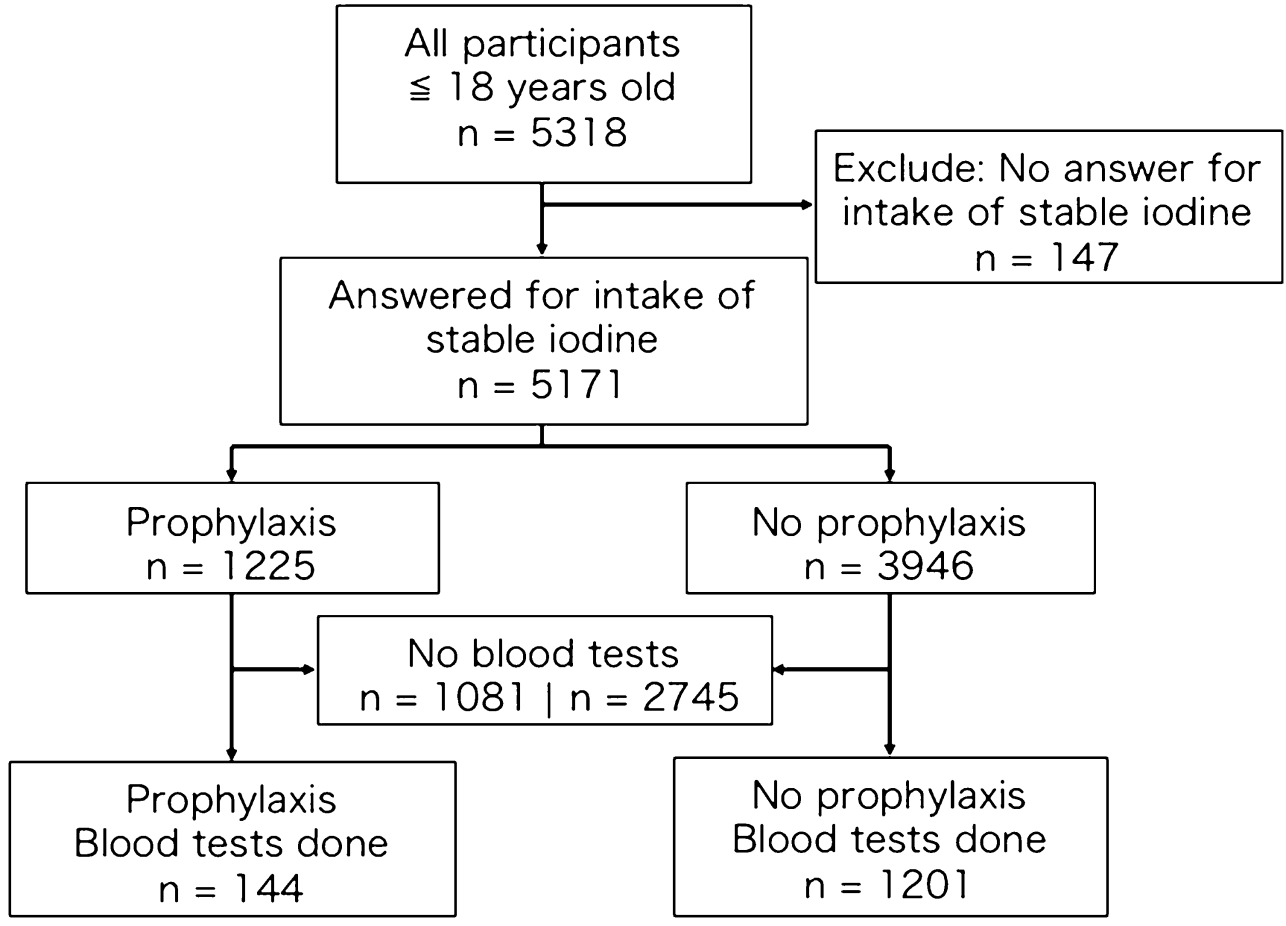
Flow diagram showing the number of participants in different outcome groups of the study

Of the total 5,318 participants (≤ 18 years old at the time of the disaster), 5,171 participants answered whether they took stable iodine after the Fukushima Disaster or not (Fig. 1). Of these, 1,225 participants underwent prophylaxis by stable iodine intake, and 3,946 did not. Among these, there were 144 and 1,201 participants who underwent blood tests in the prophylaxis and no-prophylaxis groups, respectively.

Table 1 shows the baseline characteristics of all the participants and their comparison after being stratified by stable iodine prophylaxis. Among the 5,171 participants, the numbers of males and females were 2,522 and 2,649, respectively. The mean age at the time of the disaster and the mean years since the disaster were 6.35 ± 4.11 years (mean ± standard deviation) and 3.31 ± 1.19 years, respectively. A total of 2,751 participants (53.2%) were enrolled through school screening.

**Table 1:** Participant characteristics overall and stratified by stable iodine prophylaxis

Parameter	Total	Prophylaxis	No prophylaxis	*p* value
*N*	5,171	1,225	3,946	
Female/male	2,522/2,649 (48.8/51.2)	591/634 (48.2/51.8)	1,931/2,015 (48.9/51.1)	0.697
Age at the time of screening (mean ± SD) (median (range))	9.66 ± 3.91 9 (1–24)	10.27 ± 3.26 10 (2–24)	9.47 ± 4.08 9 (1-23)	< 0.001
Age at the time of the disaster (mean ± SD) (median (range))	6.35 ± 4.11 6 (0–18)	7.09 ± 3.71 7 (0–18)	6.12 ± 4.21 6 (0-18)	< 0.001
Years since the disaster (mean ± SD)	3.31 ± 1.19	3.18 ± 1.29	3.35 ± 1.15	< 0.001
Thyroid volume (mean ± SD)	6748.84 ± 3700.34	7124.76 ± 3467.56	6631.61 ± 3762.74	< 0.001
School screening	2,751 (53.2)	1,046 (85.4)	1,705 (43.2)	< 0.001
Areas with instruction of stable iodine intake	2,013 (38.9)	1,202 (98.1)	811 (20.6)	< 0.001
Evacuation	1,984 (38.4)	517 (42.2)	1,467 (37.2)	0.002
Family history of thyroid diseases	397 (7.7)	80 (6.6)	317 (8.1)	0.095

All values are expressed as *N* (percentage), unless otherwise indicated

Data on thyroid volume were missing for 22 participants (Yes, *n* = 1; No, *n* = 21)

Data on evacuation were missing for five participants (Yes, *n* = 0; No, *n* = 5)

Data on family history of thyroid diseases were missing for 20 participants (Yes, *n* = 4; No, *n* = 16)

A comparison of the prophylaxis group with the no-prophylaxis group revealed significant differences in age at the time of screening, age at the time of the disaster, thyroid volume, evacuation, school screening, and area (*p* < 0.001) (Table 1). Among those participants who underwent blood tests, there were significant differences between the two groups with respect to age at the time of screening, evacuation, school screening, and area (*p* < 0.001) (Table 2).

**Table 2:** Comparison of participant characteristics among those with blood test data stratified by stable iodine prophylaxis

Parameter	Total	Prophylaxis	No prophylaxis	*p* value
*N*	1,345	144	1,201	
Female/Male	672/673 (50.0/50.0)	75/69 (52.1/47.9)	597/604 (49.7/50.3)	0.652
Age at the time of screening (mean ± SD)(median (range))	11.57 ± 4.29 11 (4–24)	12.37 ± 4.10 12 (6–24)	11.47 ± 4.30 10 (4–23)	0.018
Age at the time of the disaster (mean ± SD)(median (range))	8.73 ± 4.17 8 (0–18)	9.10 ± 3.98 9 (3–18)	8.68 ± 4.19 8 (0–18)	0.261
Years since the disaster (mean ± SD)	2.84 ± 0.98	3.27 ± 1.22	2.79 ± 0.94	< 0.001
Thyroid volume (mean ± SD)	8,002.73 ± 4,077.13	8,470.33 ± 3,906.08	7,946.66 (4,095.14)	0.145
School screening	43 (3.2)	24 (16.7)	19 (1.6)	< 0.001
Areas with instruction of stable iodine intake	308 (22.9)	142 (98.6)	166 (13.8)	< 0.001
Evacuation	842 (62.7)	128 (88.9)	714 (59.5)	< 0.001
Family history of thyroid diseases	142 (10.6)	20 (13.9)	122 (10.2)	0.226

All values are expressed as *N* (percentage) unless otherwise indicated.

Data on evacuation were missing for two participants (Yes, *n* = 0; No, *n* = 2)

Data on family history of thyroid diseases were missing for six participants (Yes, *n* = 0; No, *n* = 6)

There were 17 possible hypothyroidism or autoantibodies positive cases (11.8%) in the prophylaxis group and 146 cases (12.2%) in the no-prophylaxis group (Table 3). There were no possible hyperthyroidism cases in the prophylaxis group, while three cases were observed in the no-prophylaxis group. There were no statistically significant differences in the levels of TSH, FT3, or FT4 between the two groups (*p* > 0.1). Among the participants that underwent blood tests, there were no FT4 low cases in both groups. The numbers of TSH high cases were 2 (1.4%) in the prophylaxis group and 47 (3.9%) in the no-prophylaxis group. The numbers of participants with positive antibodies (TgAb and/or TPOAb) were 102 (8.5%) and 16 (11.1%) in the prophylaxis and no-prophylaxis groups, respectively.

**Table 3:** Comparison of thyroid hormones, autoantibodies, and urinary iodine levels stratified by stable iodine prophylaxis

Parameter	Total	Prophylaxis	No prophylaxis	*p* value
*N*	1,345	144	1,201	
*Continuous*				
TSH (median (range))(μIU/mL)	1.70 (0.10, 10.50)	1.79 (0.20, 6.40)	1.70 (0.10, 10.50)	0.814
FT4 (median (range))(ng/dL)	1.30 (0.80, 5.80)	1.30 (1.00, 1.70)	1.30 (0.80, 5.80)	0.91
FT3 (median (range))(pg/mL)	4.00 (2.10, 19.60)	4.05 (2.68, 5.40)	4.00 (2.10, 19.60)	0.753
Tg (median (range))(ng/mL)	16.80 (0.00, 260.20)	14.50 (0.40, 65.20)	17.10 (0.00, 260.20)	0.002
TgAb (median (range))	11.70 (10.00, 1541.00)	11.55 (10.00, 1297.00)	11.80 (10.00, 1541.00)	0.409
TPOAb (median (range))	6.80 (1.90, 600.00)	7.00 (5.00, 433.10)	6.80 (1.90, 600.00)	0.683
Urinary iodine (median (range)) (μg/L)	182.00 (25.00, 21100.00)	190.00 (25.00, 6300.00)	181.00 (25.00, 21100.00)	0.775
*Categorical*				
TSH				0.258
Low	3 (0.2)	0 (0.0)	3 (0.2)	
WNL	1293 (96.1)	142 (98.6)	1151 (95.8)	
High	49 (3.6)	2 (1.4)	47 (3.9)	
FT3				0.94
Low	1 (0.1)	0 (0.0)	1 (0.1)	
WNL	806 (59.9)	86 (59.7)	720 (60.0)	
High	538 (40.0)	58 (40.3)	480 (40.0)	
FT4				1
Low	0 (0.0)	0 (0.0)	0 (0.0)	
WNL	1,342 (99.8)	144 (100.0)	1,198 (99.8)	
High	3 (0.2)	0 (0.0)	3 (0.2)	
TgAb positive	70 (5.2)	10 (6.9)	60 (5.0)	0.426
TPOAb positive	81 (6.0)	11 (7.6)	70 (5.8)	0.498
Autoantibodies positive	118 (8.8)	16 (11.1)	102 (8.5)	0.372
TSH high or FT4 low*	49 (3.6)	2 (1.4)	47 (3.9)	0.196
TSH high and FT4 low	0 (0.0)	0 (0.0)	0 (0.0)	NA
Possible hypothyroidism** or autoantibodies positive	163 (12.1)	17 (11.8)	146 (12.2)	1
Possible hyperthyroidism (%)	3 (0.2)	0 (0.0)	3 (0.2)	1

All values are expressed as *N* (percentage) unless otherwise indicated

*WNL* within normal limit, *TSH* thyroid-stimulating hormone, *FT4* free thyroxine, *FT3* free triiodothyronine, *Tg* thyroglobulin, *TgAb* anti-thyroglobulin antibody, *TPOAb* anti-thyroid peroxidase antibody

Data on urinary iodine were missing for 127 participants (Yes, *n* = 3; No, *n* = 124)

*There were no low FT4 cases in this study.

**Possible hypothyroidism: TSH high or FT4 low

Multivariable logistic regression was used to identify factors associated with possible hypothyroidism or autoantibodies positive (Table 4). Six observations were deleted due to missing data. The analysis revealed that age at the time of the disaster, years since the disaster, male sex, school screening, and family history of thyroid diseases were factors significantly associated with possible hypothyroidism (*p* < 0.05). Intake of stable iodine was not associated with possible hypothyroidism or autoantibodies (*p* = 0.262). As for possible hyperthyroidism, multivariable analysis was not performed because there were only three cases in the no-prophylaxis group and no cases in the prophylaxis group.

**Table 4:** Multivariable logistic regression model for possible hypothyroidism (subclinical hypothyroidism, hypothyroidism, or positive for antibodies)

Parameter	OR	95% CI	*p* value
*Continuous variables*			
Age at the time of the disaster	0.96	0.92–1.00	0.034
Years since the disaster	1.19	1.02–1.40	0.029
*Categorical variables*			
Sex			
* Male*	Reference		
* Female*	1.62	1.16–2.27	0.005
School screening			
* No*	Reference		
* Yes*	2.64	1.20–5.80	0.015
Iodine prophylaxis			
* No*	Reference		
* Yes*	0.72	0.40–1.28	0.262
Family history of thyroid diseases			
* No*	Reference		
* Yes*	1.63	1.02–2.62	0.042

*OR* Odds ratio, *CI* Confidence interval

## Discussion

The present study suggests that no significant adverse effects associated with stable iodine intake on thyroid function or autoimmunity were observed over the 7-year study period. Two explanations have been suggested in our study. First, no association was observed between stable iodine prophylaxis and possible hypothyroidism using the multivariable logistic analysis. Second, the levels of thyroid dysfunction and hypothyroid-related antibodies were similar to those previously reported in the general population of children without prophylaxis [20, 21]. Additionally, there were no possible hyperthyroidism cases in the prophylaxis group. It has been shown that iodine overload results in the transient blocking of thyroid hormone synthesis through the restriction of thyroid hormone production (i.e., Wolff-Chaikoff effect) immediately after thyroid intake [21]. In the long term, it has been reported that excess iodine (e.g., supplementation, diet, or medications) is associated with thyroid dysfunction among susceptible individuals [22]. Cardiac symptoms such as tachycardia of induced hyperthyroidisms may be severe, especially in sensitive individuals such as the elderly or patients presenting with heart disease. Hypothyroidism can be an issue, particularly in adults presenting with preexisting thyroid abnormalities such as thyroiditis or thyrotoxicosis. The severity of thyroid dysfunction depends on age, situation, and daily iodine intake [1, 6, 9]. However, this study suggests that stable iodine can be safely administered to the general target population of children and adolescents for 7 years.

The present results are consistent with previous reports from Poland after the Chernobyl Nuclear incident, which suggested ﻿no statistically significant difference in TSH levels 3 years after the disaster when comparisons were made between two neonate groups (with and without prophylaxis) [8]. However, accumulating more information on possible adverse events using stable iodine will be important for future clinical preparedness. In this study, adverse effects in the following three serious situations were not evaluated. First, adverse effects among fetuses or very young children were not compiled. Since blood testing was performed for participants aged 6 years or older at the time of the screening, there was little information on very young children in this study. Second, the effects immediately after the disaster were not evaluated. The data immediately after the disaster was not available because the thyroid screening program was initiated one year after the disaster. Thus, transient adverse effects on thyroid function could not be documented. It has been reported that transient TSH increases in 0.37% of neonates after iodine prophylaxis in a Polish study [8]. Although the radiation dose, geographical location, and original iodine intake differed, there would have been some transient hypothyroidism cases who took stable iodine in Fukushima as well. Third, the effects on neurological and mental development were not evaluated. Such deleterious consequences have never been observed upon repeated use in adult rats [23, 24], in humans, or non-human primates after iodine prophylaxis [25, 26]. However, recent studies in rats have shown that repeated administration of potassium iodide during pregnancy may impair the progeny’s brain development [27]. While the current recommended use of stable iodine for children and adolescents is a single dose [1], neurological adverse effects as a possible consequence of hypothyroidism should be carefully checked to assess the possible repetitive use of stable iodine for future preparedness [25]. Hence, it also important to follow up on children who took stable iodine long after the nuclear disaster, based on clinical symptoms, and implement an appropriate thyroid screening program [28, 29].

To reduce anxiety at the time of stable iodine prophylaxis implementation, the absence of an association between stable iodine intake and possible hypothyroidism or its related antibodies should be clearly conveyed to the target population. Adverse effects of stable iodine intake have been reported as an important issue in the implementation of stable iodine [4]. Therefore, this information should be provided before a nuclear emergency occurs, since, in the case of a disaster, the time for distributing and giving instructions on intake would be limited. Japan has been revising considerations for stable iodine implementation [30]. To increase citizens’ awareness of radiation protection, a public campaign should be considered, similar to that employed in France [31]. After the Chernobyl accident, the French government initially decided to preemptively distribute stable iodine tablets to the entire population up to 10 km. In 2019, in accordance with European recommendations following the Fukushima disaster [32], the distribution areas in France were extended to 20 km. Outside these 20 km areas, France has chosen a second complementary strategy, which consists of keeping stable iodine in storage facilities for emergency distribution (more than one hundred thousand iodine tablets stored) [33]. Regardless of whether a pre-distribution or stockpile method is chosen, pharmacological information should be accurately communicated to the target population as part of a preparedness plan. At the time of a disaster, ideally, simply conveying the appropriate amount and timing of intake would be important. Therefore, our study results are beneficial in preparation for future nuclear emergencies.

This study has several limitations. First, the number of participants who developed hypothyroidism was small for analysis. With a 12% frequency of possible hypothyroidism among those without prophylaxis, an odds ratio of 1.60 was calculated with 144 exposed subjects (with prophylaxis) and 1,201 non-exposed subjects (without prophylaxis). Such an increased risk of 60% corresponds to a large increase that was unexpected just because of iodine prophylaxis. Thus, we must be careful about suggesting that there was no effect because we were only adequately powered to detect a large effect. Second, there was an unequal representation of the prophylaxis and no-prophylaxis groups, the first group accounting for 12% of the original sample (144/1,225) whereas the second group accounting for approximately one-third of the original sample (1,201/3946). Third, there were differences in participant characteristics between the prophylaxis and no-prophylaxis groups, such as age at the time of the disaster, years since the disaster, and whether the participant was evacuated. As the implementation of stable iodine prophylaxis was performed in limited areas near the Fukushima Daiichi nuclear power plant, several other differences in participant characteristics, such as age, residential area, and sense of emergency, may exist. Fourth, as blood testing was an invasive procedure and the participant was required to be accompanied by their guardian, the number of participants was limited. In the school screening, only the participants who required a secondary examination underwent the blood tests. This resulted in differences in the background characteristics between the school screening participants and other voluntary participants, which showed in the results of the multivariable regression analysis. The difference in whether school screening was done or not was based on the difference in the participant enrollment process. Moreover, there might be transient hypothyroidism cases just after iodine prophylaxis who could not be detected in this study because blood testing was performed a few years after iodine prophylaxis. Fifth, we were unable to detect ultrasonographic changes, such as diffuse thyroid hypo-echogenicity, as they could not be systematically measured in the thyroid cancer screening. In future screening, we would recommend revising the ultrasound measurement method to record such ultrasonographic changes. Sixth, this thyroid screening was performed in a single hospital, while the data included participants from more than 50 municipalities. Therefore, the results may not be representative of all areas affected by the Fukushima Nuclear Disaster. Finally, we could not consider the individual radiation dose with this dataset. It has been reported that high-dose radiation therapy causes thyroid dysfunction [34]. However, the thyroid radiation dose after the Fukushima Nuclear Disaster was estimated to be less than 100 mGy [13], which was much lower compared to the radiation dose used in such treatment procedures (more than 10 Gy as a thyroid dose) [34].

Despite its limitations, in the long term, this study would be beneficial for screening possible late adverse effects such as neurological and mental development impairment after iodine-induced prolonged hypothyroidism in young children [9]. In the case of a nuclear emergency, it would be important to follow up on the health status and clinical symptoms of children and adolescents who took stable iodine.

In conclusion, this study indicates that significant adverse effects of stable iodine intake on thyroid function were not observed among children and adolescents. This information should be clearly conveyed in preparation for future nuclear emergencies.

## Electronic supplementary material

Below is the link to the electronic supplementary material.

Supplementary file1 (DOCX 28 kb)

## Data Availability

Restrictions apply to the availability of data generated or analyzed during this study to preserve patient confidentiality or because they were used under license. The corresponding author will, on request, detail the restrictions and any conditions under which access to some data may be provided.Restrictions apply to the availability of data generated or analyzed during this study to preserve patient confidentiality or because they were used under license. The corresponding author will, on request, detail the restrictions and any conditions under which access to some data may be provided.
