# Supplementary material for: No significant association between stable iodine intake and thyroid dysfunction in children after the Fukushima Nuclear Disaster: an observational study
Source: J Endocrinol Invest. 2020 Nov 18;44(7):1491–500. doi: 10.1007/s40618-020-01454-8 (PMC8195967; doi:10.1007/s40618-020-01454-8)
Supplement: Supplementary file 1 — Supplementary file1 (DOCX 28 kb) [file 40618_2020_1454_MOESM1_ESM.docx]

No Significant Association Between Stable Iodine Intake and Thyroid Dysfunction in Children After the Fukushima Nuclear Disaster: An Observational Study

*Journal of Endocrinological Investigation*

Yoshitaka Nishikawa, Chiaki Suzuki, Yoshimitsu Takahashi, Toyoaki Sawano, Hirokatsu Kinoshita, Enora Clero, Dominique Laurier, Guillaume Phan, Takeo Nakayama, Masaharu Tsubokura

Corresponding author: Yoshitaka Nishikawa, MD, PhD. Department of Internal Medicine, Hirata Central Hospital, 4, Shimizu-uchi, Kami-Yomogida, Hirata-mura, Ishikawa-gun, Fukushima 963-8202, Japan

TEL +81-247-55-3333, E-mail: ynishikawa-tky@umin.ac.jp

Supplemental Table. Higher and lower limits for thyroid hormone and thresholds for positive autoantibodies.

Thyroid disorders were diagnosed depending on the results of the blood tests and the guidelines for the diagnosis of thyroid disease, published by the Japan Thyroid Association [19]. For TSH, the lower limit was documented as 0.1 ng/dL. For other parameters, higher and lower limits shown on the website of BML, Inc. were used in this study (<http://uwb01.bml.co.jp/kensa/pdf/BML2016-4.pdf>).

| Until 2016/3/31 | Lower limit | Higher limit |
| --- | --- | --- |
| TSH | 0.1 μIU/mL | 4.0 μIU/mL |
| FT4 | 0.8 ng/dL | 1.9 ng/dL |
| FT3 | 2.2 pg/mL | 4.1 pg/mL |
| From 2017/4/1 | Lower limit | Higher limit |
| TSH | 0.1 μIU/mL | 5.0 μIU/mL |
| FT4 | 0.9 ng/dL | 1.7 ng/dL |
| FT3 | 2.30 pg/mL | 4.0 pg/mL |
|  | Normal range | Positive |
| TgAb | <28.0 IU/mL | ≧28.0 IU/mL |
| TPOAb | <16.0 IU/mL | ≧16.0 IU/mL |

TSH, Thyroid-stimulating hormone; FT4, Free thyroxine; FT3, Free triiodothyronine;

TgAb, Anti-thyroglobulin antibody; TPOAb, Anti-thyroid peroxidase antibody.
